# Experimental and theoretical insights into LDH based on iron for photoelectrochemical water splitting

**DOI:** 10.1038/s41598-025-17648-0

**Published:** 2025-10-14

**Authors:** Fatma Mohamed, Omnia M. Salem, Khaled Abdelkarem, Mohamed Shaban, Ashour M. Ahmed

**Affiliations:** 1 Nanophotonics and Applications Lab, Faculty of Science, Beni-Suef University, Beni-Suef, 62514 Egypt; 2 Materials Science Research Laboratory, Chemistry Department, Faculty of Science, Beni-Suef University, Beni-Suef, Egypt; 3 Department of Physics, Faculty of Science, Islamic University of Madinah, P. O. Box: 170, AlMadinah Almonawara, 42351 Saudi Arabia; 4 Physics Department, College of Science, Imam Mohammad Ibn Saud Islamic University (IMSIU), Riyadh, 11623 Saudi Arabia; 5 Department of Physics, Chonnam National University, Gwangju, 61186 Republic of Korea

**Keywords:** Layered double hydroxide, LDH, Mg/Fe, Ca/Fe, Water splitting, Catalytic efficiency, DFT., Electrochemistry, Energy, Theoretical chemistry, Renewable energy

## Abstract

This study presents a novel layered double hydroxide **(**LDH) composite consisting of Mg/Fe and Ca/Fe-LDH layers, synthesized via a co-precipitation method, aimed at enhancing hydrogen production from solar energy. The composites have bandgaps of 2.01 eV and 2.81 eV, respectively, making them ideal for photoelectrochemical (PEC) water splitting. The study demonstrates that using Mg/Fe-LDH can significantly increase the rate of hydrogen generation, achieving a catalytic H_2_ production rate of 2542.36 mmol/h·cm^2^ and a catalytic efficiency of 56.39% at 460 nm and 59.85% at 490 nm. ABPE achieves maximum peaks of 5.75% at 0.92 V and 5.33% at 0.4 V, setting new standards for LDH-based photocatalysts. The research also examines the effects of temperature and monochromatic light on PEC performance, thermodynamic parameters, electrochemical surface area, Tafel slopes, and hydrogen evolution kinetics. The LDH photoelectrode’s stability and reusability reveals its long-term application potential, providing fresh insights on improving PEC methods for industrial-scale water splitting in sunlight. Also, this is further corroborated by Density Functional Theory (DFT). This distinctive methodology was selected for the design of LDH nanomaterials for hydrogen-generation applications.

**Supplementary Information:**

The online version contains supplementary material available at 10.1038/s41598-025-17648-0.

## Introduction

Owing to the huge decline of fossil fuels and the associated environmental issues brought on by human activity and economic growth, much effort has been made to find unlimited and environmentally acceptable energy sources to replace traditional fossil fuels. Consequently, the sustainable development of clean energy can successfully address the issue of environmental contamination, which is seen as a major concern^1,2^. It is common knowledge that solar energy is one of the cleanest and safest energy sources and that its robust development may significantly reduce environmental pollution. To optimize solar energy resources for water splitting and hydrogen production, the electrochemical water splitting process powered by sustainable energy has been identified as a highly promising alternative to the existing hydrocarbon economy^3^. Photoelectrochemical (PEC) water-splitting technology is a promising method for converting solar energy into sustainable hydrogen fuel. Many semiconductors with non-noble metals were recommended for large light absorption and H_2_ generation, among other materials. Layered double hydroxides (LDHs) offer numerous advantages as catalysts in photoelectrochemical (PEC) water splitting, enhancing the PEC activity of wide-bandgap semiconductors^4–6^. They can diminish charge recombination and enhance the kinetics of the water oxidation reaction. Moreover, their elevated layer charge density and unique electrical Characteristics. LDHs consist of divalent and trivalent metal cation layers that are covered with hydroxide ions^7–9^. In particular, the synergistic impact was fundamentally amplified by the existence of flexible chemical structures, a greater number of exposed active sites on the layered surface, and an acceleration of the photocatalytic hydrogen evolution reaction^10,11^. Currently, the use of bifunctional electrocatalysts is a highly promising approach in the field of industrial hydrogen production. Combining various efficient electrocatalysts into a hierarchical framework to create bifunctional catalysts has the potential to streamline the development and design of electrolytic devices while also decreasing operational expenses^12,13^. Layered double hydroxides (LDHs) exhibit favorable characteristics for photocatalysis and are regarded as superior alternatives to TiO_2_-based photocatalysts because of their layered structure, compositional flexibility, controlled dimension, cost-effectiveness, and straightforward preparation methods^14^. Of the many LDHs, the characteristics of Fe-based LDHs like Ni-Fe-LDH include easy production, low-cost growth, a high specific surface area, and a dense concentration of active centers that enhance interaction with various catalytic materials, making them very attractive. Harvesting these distinct pathways of layered double hydroxides and depositing Ni-Fe layered double hydroxides can enhance their electroactivity^15–18^. Iron metal hydrolysis for on-board hydrogen generation is gaining significant interest because of its quick and uncomplicated nature^19^. High quantities of NaOH and alloying Fe with common transition metals (Fe and Cu), alkaline earth metals (Ca and Mg), and other electrochemically noble metals (Ga, Li, and Bi) are applied to improve the hydrolysis reaction to alleviate this problem. The creation of Fe(OH)_3_ and Mg(OH)_2_ photocatalysts impedes hydrolysis reactions^19^, and reusing solid hydrolysis products may be challenging because of the presence of doped metals. This issue has been overlooked in literature, and it is evident that this practice is not linked with the concepts of green and sustainable development. This work rigorously examines the influence of various cations in LDH on the efficiency of photoelectrochemical (PEC) water splitting. Magnesium ferrite layered double hydroxide (Mg/Fe-LDH) and calcium ferrite layered double hydroxide (Ca/Fe-LDH) were produced using a straightforward and economical co-precipitation technique. LDH was comprehensively assessed using energy dispersive X-ray spectroscopy (EDXS), UV-vis spectroscopy, scanning electron microscopy (FE-SEM), and X-ray diffraction (XRD). The performance of LDHs was assessed using the incident-photon-to-current efficiency (IPCE) and bias potential conversion (ABPE). In addition, the stability, reusability, and the amount of hydrogen moles.

## Synthesis of LDHs

### Synthesis of Mg/Fe-LDH

A 1:1 molar ratio of Mg/Fe-LDH was synthesized via co-precipitation. Iron sulphate (0.1 M) and magnesium nitrate (0.1 M) were dissolved in 100 ml of H_2_O before the pH was adjusted with 2 N NaOH applied dropwise at 60 °C while swirling vigorously until the pH reached 10. The LDH that was created after 24 h of stirring was collected, washed many times with tepid distilled water until the pH reached 7, and subsequently dried overnight at 50 °C.

### Synthesis of Ca/Fe-LDH

Ca/Fe-LDH with a molar ratio of 1:1 was synthesized utilizing the co-precipitation technique. A solution of calcium nitrate (0.1 M) and iron nitrate (0.1 M) was made by dissolving them in 100 ml of water. The pH of the solution was adjusted to 10 by gradually adding 2 N NaOH solution while rapidly swirling at a temperature of 60 °C. After being stirred for 24 h, the generated LDH was collected and treated with numerous washes using warm distilled water until attaining a pH of 7. It was then dried at a temperature of 50 °C overnight^17,20^.

### Theoretical study

A deep analysis of the Ca/Fe and Mg/Fe-LDH at the molecular level will reveal the causes for the structure-activity relationship. This part exhibited the improvement of HER activity on the Ca/Fe and Mg/Fe-LDH by simulating the geometric structures and electrostatic potential characteristics using DFT. The Gaussian 09 software suite was utilized to conduct the chemical computations outlined in this study, and drawing structures of Ca/Fe-LDH and Mg/Fe-LDH by Gauss view and VESTA illustrated in Fig. 10 (a and d), then take one Molecule for Study DFT and electrostatic potential(ESP). Computational data is produced via the density functional theory (DFT) method with the B3LYP/6-31G(d, p) model chemistry, and illustrated SCF and unit cell Parameters in Tables S1 and S2, respectively. Density functional theory (DFT) approaches have been employed utilizing the B3LYP functional and the 6-311G basis set. These approaches have been selected to determine the band gap of the species under inquiry, as they have been successfully implemented in analogous systems. DFT calculations yield data on the energy gap (E_gap_ = E_LUMO_ − E_HOMO_), facilitate the identification of the molecular electrostatic potentials (MEP) plot, which visualizes the whole density, and delineate the highest occupied and lowest unoccupied molecular orbitals, E_HOMO_ and E_LUMO_, respectively.

### Characterization of LDH photocatalysts

The produced photocatalysts underwent several characterisation procedures to examine their properties. The crystal structure and phase purity were measured using X-ray diffractometry (XRD, Rigaku D/Max 2500). The XRD investigation was conducted at 30 mA and 40 kV, covering a scan range from 5 to 80°. The morphology of the photocatalysts was studied using a field emission scanning electron microscope (FE-SEM, Zeiss Sigma 500 VP). To assess the chemical composition, energy-dispersive X-ray (EDXS) spectroscopy was done in conjunction with the FE-SEM. Fourier transform infrared spectroscopy (FTIR, NICOLET 6700) was performed to analyze the functional groups contained in the produced materials. The optical characteristics were studied using a double-beam spectrophotometer (PerkinElmer Lambda 950) in the range of 200–1000 nm. 3D image created by ImageJ software, and the roughness was determined by Gwyddion.

### Electrodes Preparation and electrochemical measurements

The electrode fabrication process began with the cleaning of the graphite substrate using methanol and ethanol. Approximately 2.0 mg of the LDH photocatalyst was then mixed with 0.20 ml of a 5 wt% Nafion solution and 0.40 ml of isopropanol. This mixture underwent 120 min of ultrasonic treatment to achieve a homogeneous suspension. The 1 mg of the resulting suspension was loaded onto a graphite sheet and dried at 50 °C to create the electrode. The assessment of hydrogen gas generation was conducted in a 0.3 M Na_2_SO_3_ aqueous electrolyte solution with a pH of 7.0. For the photoelectrochemical (PEC) studies, a two-electrode OrigaFlex potentiostat (OrigaLys ElectroChem) was utilized. The experimental setup included a solar simulator equipped with an Xe-lamp emitting light (AM 1.5 G, 100 mW/cm^2^). A platinum electrode is used as the auxiliary electrode to evaluate the photoelectrode’s catalytic activity. The working electrode employed was the fabricated LDH/graphite electrode. To record the photocurrent density (J_ph_) responses, a scanning voltage (V) ranging from ‒1 to + 1 V was applied at room temperature. The photocurrent density was measured under dark conditions as well as under the illumination of the white Xe lamp and monochromatic light.

## Results and discussion

The ionic radii, the most common oxidation states, and electronegativity for Mg, Ca, and Fe can take different values. The ionic radius of Fe(II), Fe(III), Ca (II), and Mg (II) is 70 pm, 60 pm, 100 pm, and 72 pm, respectively. According to the Pauling scale, the electronegativity of Fe, Mg, and Ca atoms is 1.83, 1.31, and 1.00, respectively. These factors affect the number of carrier charges, structure morphology, and crystallite size and therefore modify the chemical and physical properties of photocatalysts^21,22^.

### FE-SEM and surface roughness analysis

**Fig. 1:**
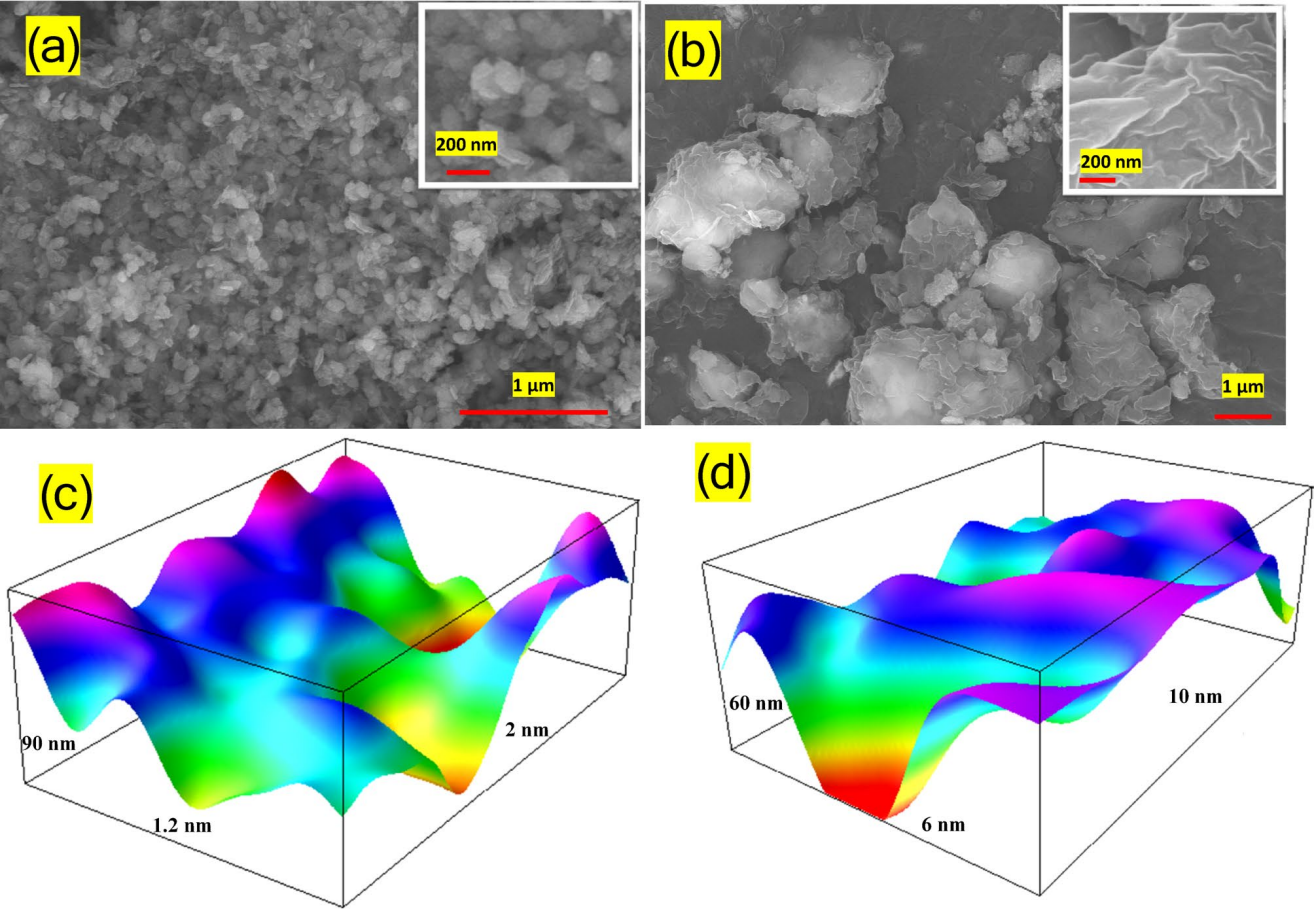
FE-SEM image of surface morphology of (**a**) Mg/Fe-LDH and (**b**) Ca/Fe-LDH, used ImageJ software 3D surface roughness for (**c**) Mg/Fe-LDH and (**d**) ؤCa/Fe-LDH .

The morphologies of Ca/Fe and Mg/Fe-LDH are presented in Fig. 1. The Ca/Fe-LDH nanomaterial has a platelet-like structure with rough flake fragments on its surface (Fig. 1(a)). There are a large number of nanoplatelets with very small sizes. The range of nanoplatelet sizes is from 36 up to 179 nm. The FE-SEM characterization of Mg/Fe-LDH photocatalyst shows agglomerated large particles composed of a well-developed layered structure with sheet-like structures (Fig. 1(b)). These sheets have variable shapes and sizes. Also, the surface of the sheets is wavy and wrinkled, which indicates a large surface area with an average size of 103 nm. Figure 1 (c) and (d) illustrate the surface roughness measurements of Ca/Fe and Mg/Fe-LDH as analyzed using Gwyddion software, and a 3D image using ImageJ software. The roughness of Mg/Fe-LDH is higher than that of Ca/Fe due to the large number of small-sized nanoplatelets. The Ca/Fe surface roughness decreased due to the agglomeration and the formation of large nanoplates^21,23^. The root-mean-square (RMS) roughness, written as R_q_, is often used to measure the height difference. Mg/Fe-LDH has an RMS value of 7.98 nm, and Ca/Fe-LDH has an RMS value of 3.5 nm. Depending on the surface roughness of the Mg/Fe-LDH nanomaterial, which is rougher than that of the Ca/Fe-LDH nanomaterial, this led Mg/Fe-LDH has high performance in hydrogen evolution reactions (HER), and it has better catalytic performance than Ca/Fe-LDH. This enhanced activity can be attributed to the increased surface roughness, which inherently provides a greater number of active sites for catalysis^24–26^. The Mg/Fe-LDH nanomaterial’s complex network and the rougher surface are one of the main reasons why it has better electrocatalytic properties for making hydrogen more efficient^24,27,28^.

### XRD analysis

**Fig. 2:**
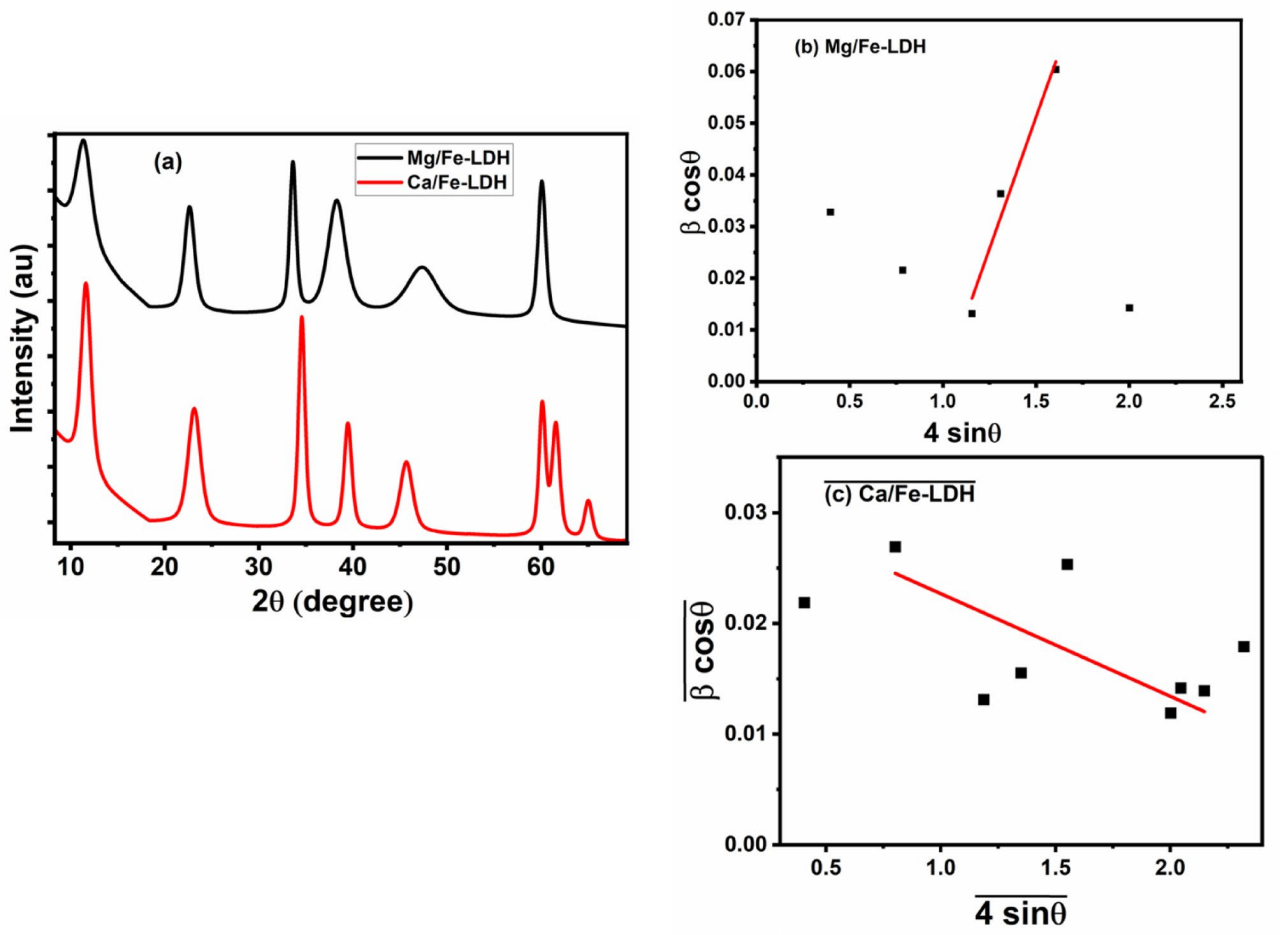
(**a**)XRD pattern, (**b**) W-H plot of Mg/Fe-LDH, and (**c**) W-H plot of Ca/Fe-LDH.

Figure 2(a) depicts the X-ray diffraction (XRD) patterns of the Mg/Fe-LDH and Ca/Fe-LDH photocatalysts. The XRD analysis confirms the presence of the LDH structure in both photocatalysts, as evidenced by distinct peaks in their XRD patterns. These LDH photocatalysts exhibit well-crystallized characteristics and possess a hexagonal structure. In the case of Ca/Fe-LDH, the observed peaks at 2θ = 11.62, 23.12, 34.56, 45.48, 60.08, and 61.52 degrees correspond to the (003), (006), (114), (027), (112), and (113) reflections, respectively. These peak positions align with previous studies conducted by^29–31^. It is worth noting that the photocatalysts also contain some Ca species, such as Ca(OH)_2_ and CaO impurities. Similarly, for Mg/Fe-LDH, the observed peaks at 2θ = 11.38, 22.62, 33.59, 38.25, 47.33, and 60.03 degrees can be attributed to the (003), (006), (009), (015), (018), and (110) reflections, respectively. These peak positions align with previous studies^32–34^. The XRD peaks Mg/Fe-LDH and Ca/Fe-LDH are mostly from Mg /Fe-LDH JCPDS (#96-153-7460) and for JCPDS Ca/Fe-LDH (#96-434-0356) and (#96-901-3961) according to crystallography open database (COD). To determine the crystallite size (D) and microstrain (ɛ) of the photocatalysts, the Williamson-Hall (W-H) model was utilized. The W-H model is represented by the following Eq(1).^32,35–37^:

1 $$\:{{\upbeta\:}}_{\:}\:\text{c}\text{o}\text{s}\left({\uptheta\:}\right)=\frac{\text{k}\:{\uplambda\:}}{{\text{D}}_{\:}\:}+4{\:{\upepsilon\:}}_{\:}\:\text{s}\text{i}\text{n}\left({\uptheta\:}\right)$$

where k represents the shape factor for photocatalysts, λ denotes the XRD wavelength, and β corresponds to the broadening of the diffraction lines in radians. Figure 2(b) and(c) and Table 1 provide comprehensive details of the data and the W-H plot. In the W-H plot, the x-axis represents the values of 4 sin(θ), while the y-axis displays β cos(θ). A linear fit was applied to the plot to extract the crystallite size (from the y-intercept) and strain (from the slope). Microstrain refers to local lattice distortions or disorders within the crystal structure, which can arise from defects or impurities present in the crystal. The low microstrain observed suggests minimal distortion within the crystal lattice. Table 1 reveals that the crystallite sizes of Mg/Fe-LDH and Ca/Fe-LDH are 87.0 nm and 27.7 nm, respectively. Moreover, Mg/Fe-LDH (28 × 10^‒3^) exhibits a higher microstrain compared to Ca/Fe-LDH (25 × 10^‒3^). The negative strain observed in the Mg/Fe-LDH photocatalyst indicates lattice shrinkage.

**Table 1:** Parameters of W-H model.

	Mg/Fe-LDH	Ca/Fe-LDH
Crystallite size (nm)	87.00	27.76
Microstrain	0.028	0.025

### FTIR analysis

**Fig. 3:**
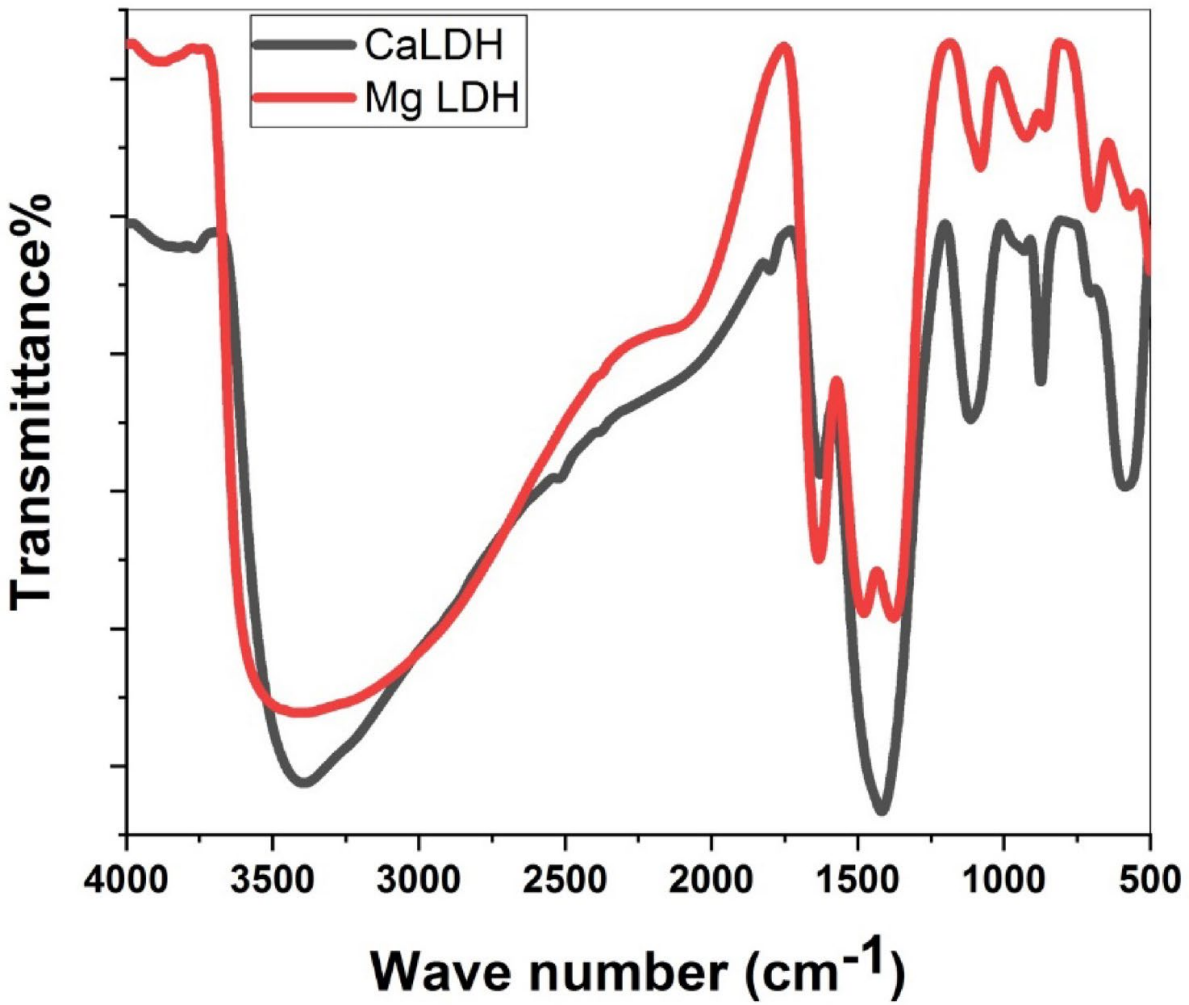
The FTIR spectra for Ca/Fe-LDH and Mg/Fe-LDH.

The FTIR spectra of the Mg/Fe-LDH and Ca/Fe-LDH photocatalysts displayed different absorption peaks. Notably, Mg/Fe-LDH revealed absorption bands at particular wavenumbers: broad bands between 3100 and 3600 cm^− 1^ were ascribed to the metallic hydroxide layer’s ‒OH stretching and interlayer water molecules^38^. Additionally, a strong band at 1377.99 cm^− 1^ indicates the existence of NO_3_ anions in the interlayer area, signifying carbonate groups, and 480 cm^‒1^, suggestive of metal-oxygen vibrations (Fig. 3). The appearance of these absorption peaks clearly supports the presence of important functional groups within the photocatalyst structure^39^. These functional groups contain hydroxyl groups, carbonates, and metal-oxygen linkages, A related observation was made in the FTIR spectra of Ca/Fe-LDH photocatalyst, which revealed analogous absorption peaks (Fig. 3). This closeness in functional groups emphasizes the potential universality in the interaction mechanisms between these nanoparticles and plant systems^40^.

Notably, all LDH photocatalysts revealed common absorption bands in the FTIR spectra, including a broad absorption band in the 3300–3500 cm^‒1^ area that was ascribed to the OH stretching mode of the interlayer water and basal layer. The bending mode of interlayer water and anion groups was also detected as a band in the 1620–1650 cm^‒1^ range. Additionally, a unique band associated with MO and MOH vibrations was identified in the 400–800 cm^‒1^ range^41^.

### EDXS analysis

**Fig. 4:**
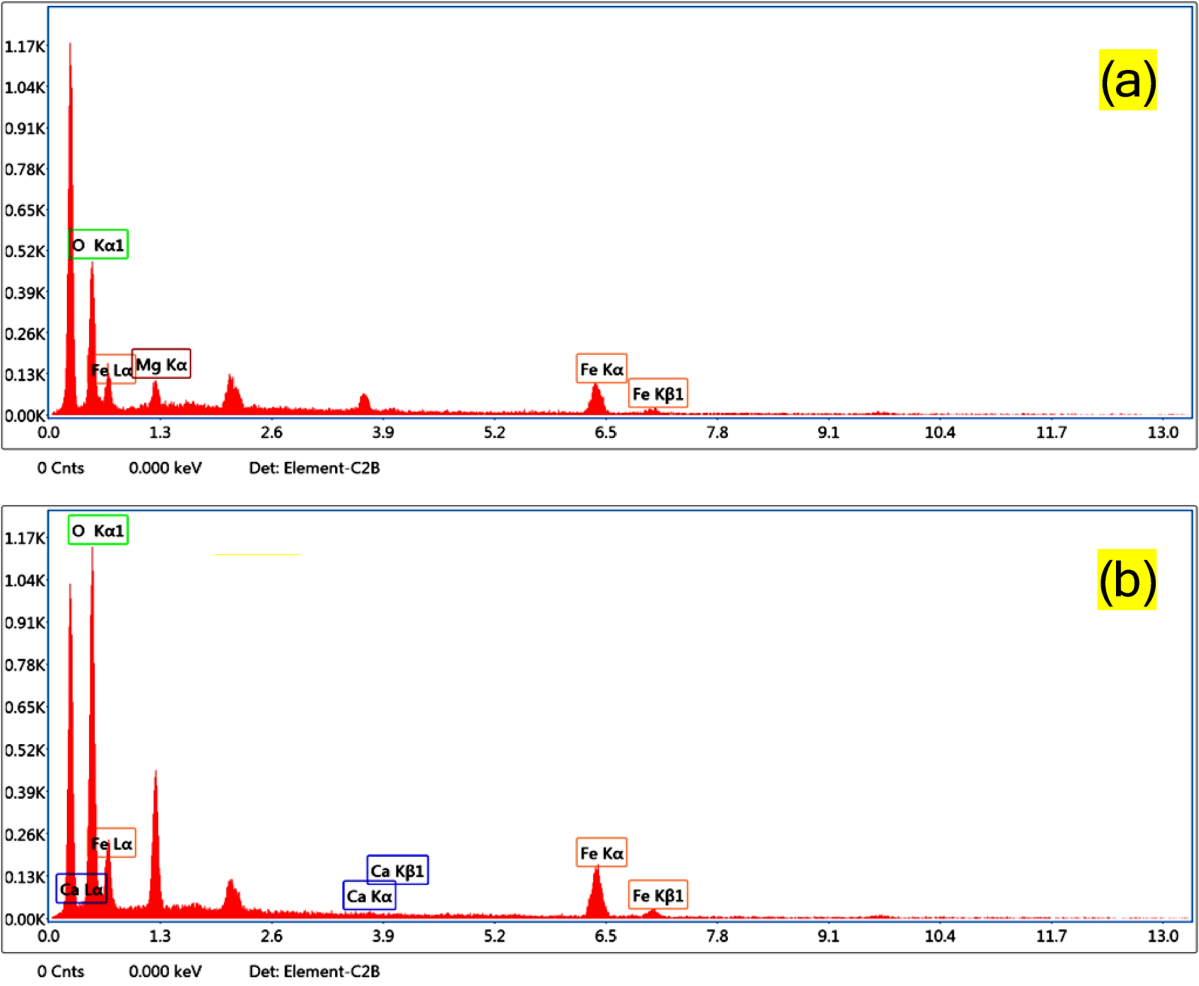
EDXS spectra of (**a**) Mg/Fe-LDH and (**b**) Ca/Fe-LDH.

A photocatalyst’s elemental composition and chemical analysis can be detailed using the analytical approach known as energy-dispersive X-ray spectroscopy (EDXS)^42^. The EDXS spectrum shown in Fig. 4 (a, b) indicates the presence of iron, magnesium, oxygen, and calcium elements. No impurity signals were detected in the EDXS spectra, which matches the XRD results. The EDXS analysis supported presence of the intended cations of Mg/Fe-LDH and Ca/Fe-LDH. and confirmed Success synthesis of them by XRD and FTIR analysis.

### UV-vis spectroscopy analysis

**Fig. 5:**
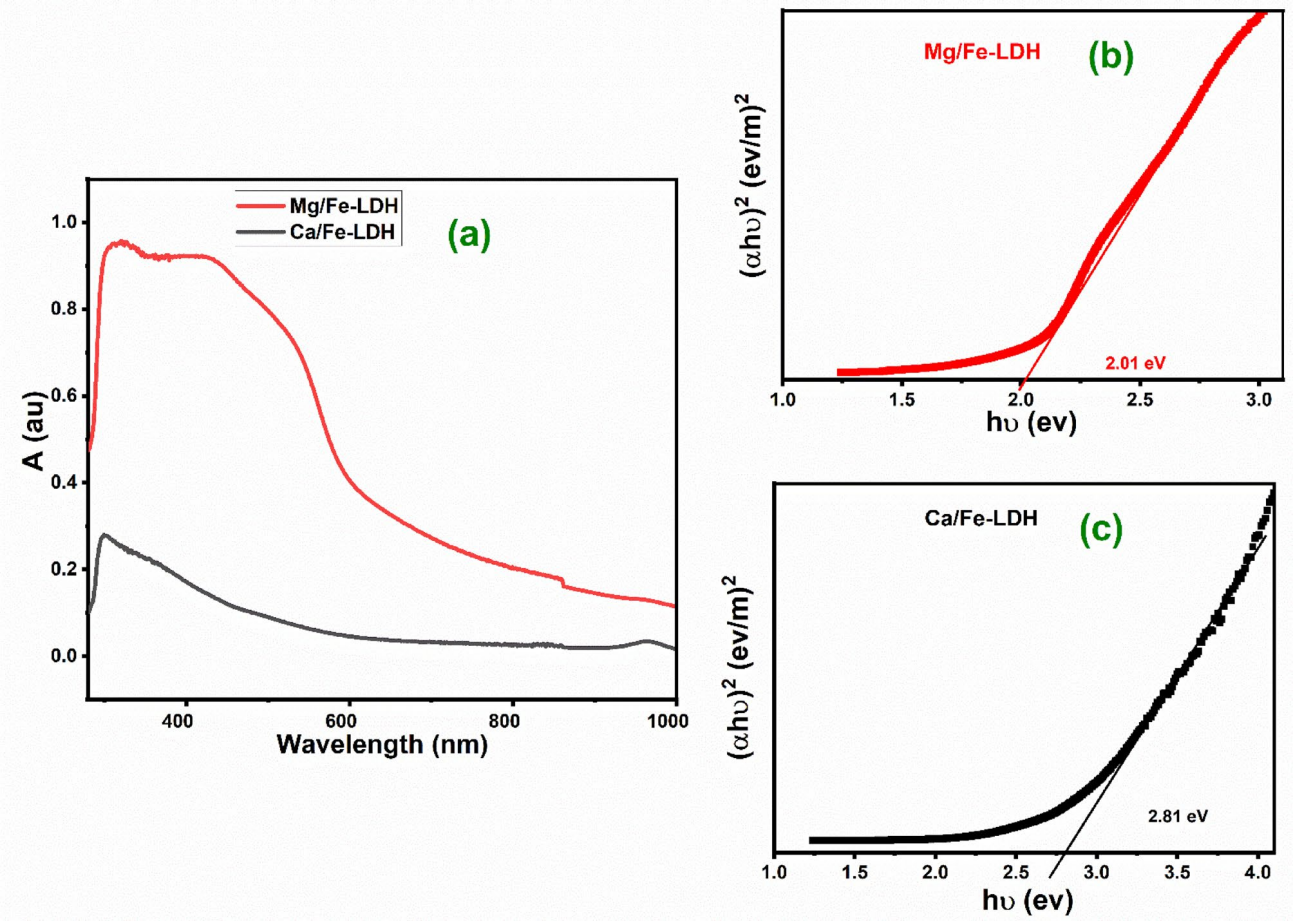
(**a**) Uv-Vis spectra and (**b**, **c**) Tauc curves for Mg/Fe-LDH and Ca/Fe-LDH.

The absorption spectra of LDH had a significant impact on the PEC performance. The visible region, with wavelengths ranging from 400 to 600 nm, is critical for photocatalytic applications due to its dominance in solar irradiation^43^. The absorbance spectra of Mg/Fe-LDH and Ca/Fe-LDH as a function of wavelength from 200 to 1000 nm are displayed in Fig. 5(a). Figure 5(b) (red line) shows the Mg/Fe-LDH photocatalyst has a broad and intense absorption band. The Ca/Fe-LDH photocatalyst presented an absorption band within the UV region, as seen in Fig. 5(c) (black line). Above 350 nm, the absorption intensity of the Ca/Fe-LDH photocatalyst decreased. This indicates the Ca/Fe-LDH has very low photo-absorption in the visible region. The absorption edge of Mg/Fe-LDH shifts towards the higher wavelength side (visible region) compared to Mg/Fe-LDH. Also, Mg/Fe-LDH absorbs more photons in the UV and visible regions than Ca/Fe-LDH. Mg/Fe-LDH has two primary peaks at about 320 and 400 nm. The large peak at 386 nm and the weak peak at roughly 320 nm is attributable to π-π* transition of C = C. The faint absorption peak that emerges at 400 nm is due to n-π* concerning the Mg/Fe-LDH^44^. This suggests a decrease in the band gap energy, which can lead to more electron/hole pair generation under visible light irradiation, resulting in improved photocatalytic hydrogen activity. The optical bandgap energy for direct transition can be assessed by using the Tauc Eqs as illustrated in eq(2).^45–47^:

2 $$\:{\left({\upalpha\:}\:{\text{E}}_{\text{p}\text{h}}\right)}^{2}\:=\:\text{K}\:({\text{E}}_{\text{p}\text{h}}-\:\text{E}\text{g}{)}^{\:}$$

where $\:{\upalpha\:}$ is the absorption coefficient, $\:{\text{E}}_{\text{p}\text{h}}$ is the energy of an incident photon, $\:\text{E}\text{g}$ is the energy bandgap, and K is the constant. Using Fig. 5(b, c), the $\:\text{E}\text{g}$ value of the Mg/Fe-LDH (2.01 eV) is low compared to the Ca/Fe-LDH ($\:\text{E}\text{g}$ = 2.81 eV) value. The decrease in the bandgap of Mg/Fe-LDH agrees with data obtained from the crystalline nature and nanostructured morphology as observed in the FE-SEM and XRD analyses. The electronic modification led to a high absorption spectrum and a decreased band gap^48–50^. This indicates that Mg/Fe-LDH is suitable for solar energy applications.

### Photoelectrochemical (PEC) measurements

#### The PEC behavior of LDH photoanodes

**Fig. 6:**
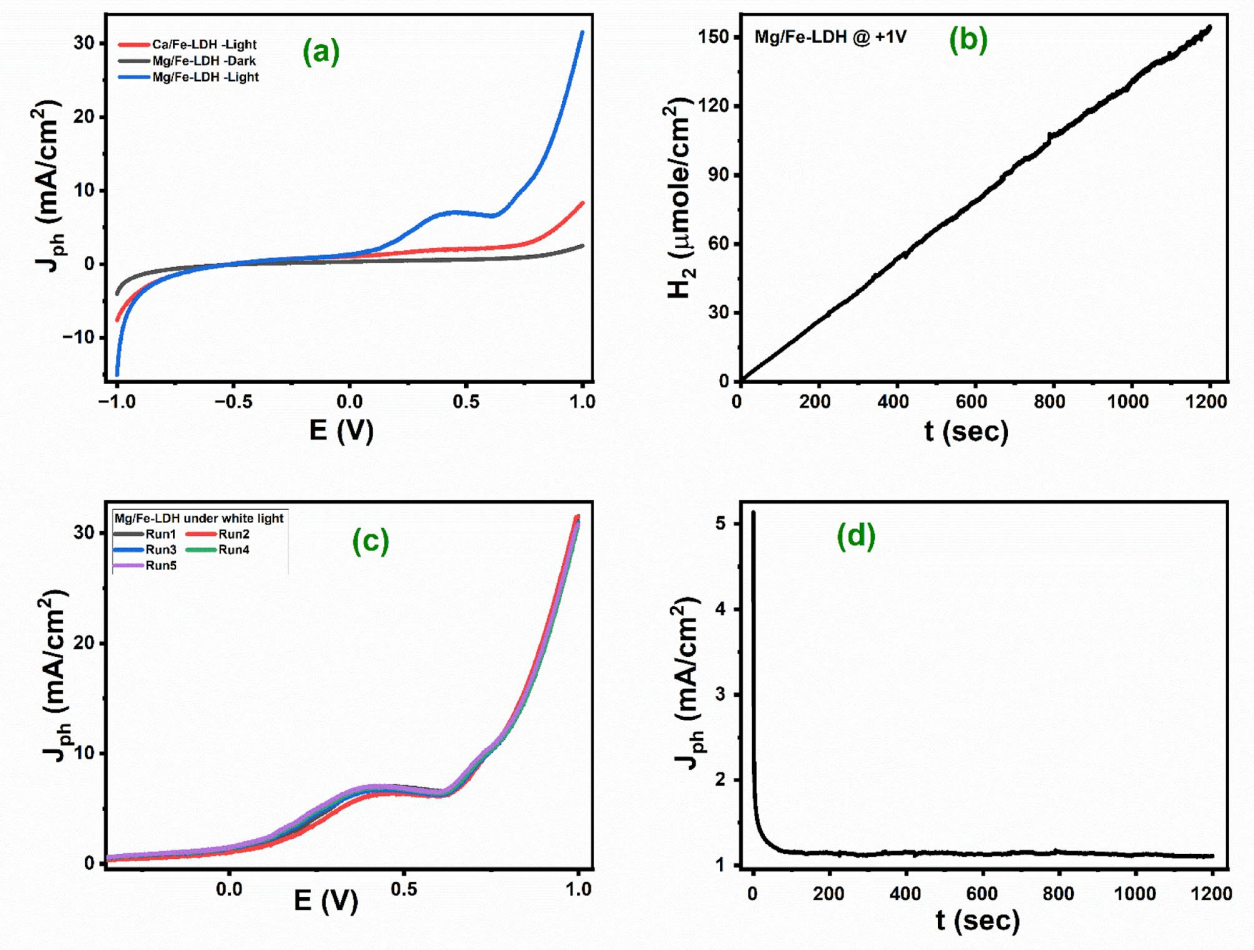
Under white light illumination (**a**) Jph-E curves in dark and light for two photoelectrodes (Mg/Fe-LDH & Ca/Fe-LDH); (**b**) The H_2_ moles evaluation for the Mg/Fe-LDH photoelectrode at room temperature; (**c**) Reproducible studies of Mg/Fe-LDH photoelectrode, and (**d**) stability Curve of the Mg/Fe-LDH.

Potentiometry, cyclic voltammetry, and amperometry measurements were performed in the 0.3 M Na_2_SO_3_ electrolyte under illumination of white and monochromatic lights to evaluate the photoelectrode’s catalytic activity. Figure 6(a) shows the Jph-E curves of photoanodes in the cases of dark and white lighting at A range of voltages from − 1 to + 1 V. The typical curves for photoelectrochemical current density (Jph) and potential (E) reveal that the maximum Jph values may be seen in the positive voltage range. This indicates that the electrodes are used as photoanodes^51^. When compared to the Ca/Fe-LDH photoanode, the photoactivity of the Mg/Fe-LDH photoanode is much higher. This shows that the combination of iron and magnesium has a substantially greater impact on the photocatalyst performance than iron and calcium LDH. In light of existence, the photocurrent density for the Mg/Fe-LDH photoanode, which is 31.55 mA/cm^2^ at 1 V, is almost 3.9 times higher than the photocurrent density for the Ca/Fe-LDH photoanode, which is about 8.2 mA/cm^2^ at 1 V. The activity of the Mg/Fe-LDH in the dark was less than in the case of light exposure, with a maximum current of around 2.49 mA/cm^2^ at 1 V. This can be attributed to several factorsPEC performance is substantially influenced by the photoanode material’s band gap energy. A reduced band gap permits the material to absorb a greater range of the solar spectrum, making it more efficient at converting light into electrical energy. A semiconductor photocatalyst utilized in a PEC water-splitting cell requires to have an optimum band gap of about 2.0 eV^52^. Mg/Fe-LDH has a smaller band gap of 2.01 eV than Ca/Fe-LDH at 2.81 eV; that’s why it is much better at harvesting solar energy. Additionally, efficient charge separation and transport are essential for high PEC performance. Mg/Fe-LDH has superior charge separation and transport properties than Ca/Fe-LDH due to its electronic structure and charge carrier mobility, resulting in better photoconversion efficiency^53,54^. The modified Mg/Fe-LDH thin photocatalysts that are located on the surface of the Mg/Fe-LDH photoanode contribute to an increase in the estimated carrier concentration^32,55^. This is because Mg/Fe-LDH is reliant on photogenerated holes occupying the surface of the photoanode to function properly. The oxidation of water is increased as a result, which results in a greater release of hydrogen^56,57^. This indicates that the hydrogen evolution reaction’s (HER) kinetics are improved upon the deposition of Mg/Fe-LDH. Based on the J-time (amperometry measurements), the following Eq. (3) was applied to determine the total number of moles of hydrogen produced by the process of photoelectrochemical water splitting^58^.

3 $$\:\text{N}\text{u}\text{m}\text{b}\text{e}\text{r}\:\text{o}\text{f}\:\text{h}\text{y}\text{d}\text{r}\text{o}\text{g}\text{e}\text{n}\:\text{m}\text{o}\text{l}\text{e}\text{s}=\:{\int\:}_{0}^{\text{t}}\frac{\text{J}\text{p}\text{h}}{\text{F}}\:\text{d}\text{t}$$

where Jph is photocurrent density, F is the Faraday constant (96,500 C/mol), and t is the period. Figure 6(b) depicts this relationship between the number of hydrogen moles and time. The estimated hydrogen output rate was about 2542.36 µmole/h.cm^2^.

The consistency and stability of the Mg/Fe-LDH photoelectrode are examined regarding the total number of runs (5 runs). The results are presented in Fig. 6(c), which demonstrates that the Mg/Fe-LDH maintains 97.46% photocurrent even after five runs have been completed. This shows very clearly that the optimized LDH photocatalyst, Mg/Fe-LDH, is very stable and can be used as a photoanode for a long time in hydrogen production. A chronoamperometric current density vs. time curve was utilized to investigate the stability of the Mg/Fe-LDH, revealing a constant photocurrent density of 0.5 mA cm^− 2^ sustained for 1200 s at 1 V. As illustrated in Fig. 6 (d), this supports the endurance of the Mg/Fe-LDH and shows its potential for the long term.

**Fig. 7:**
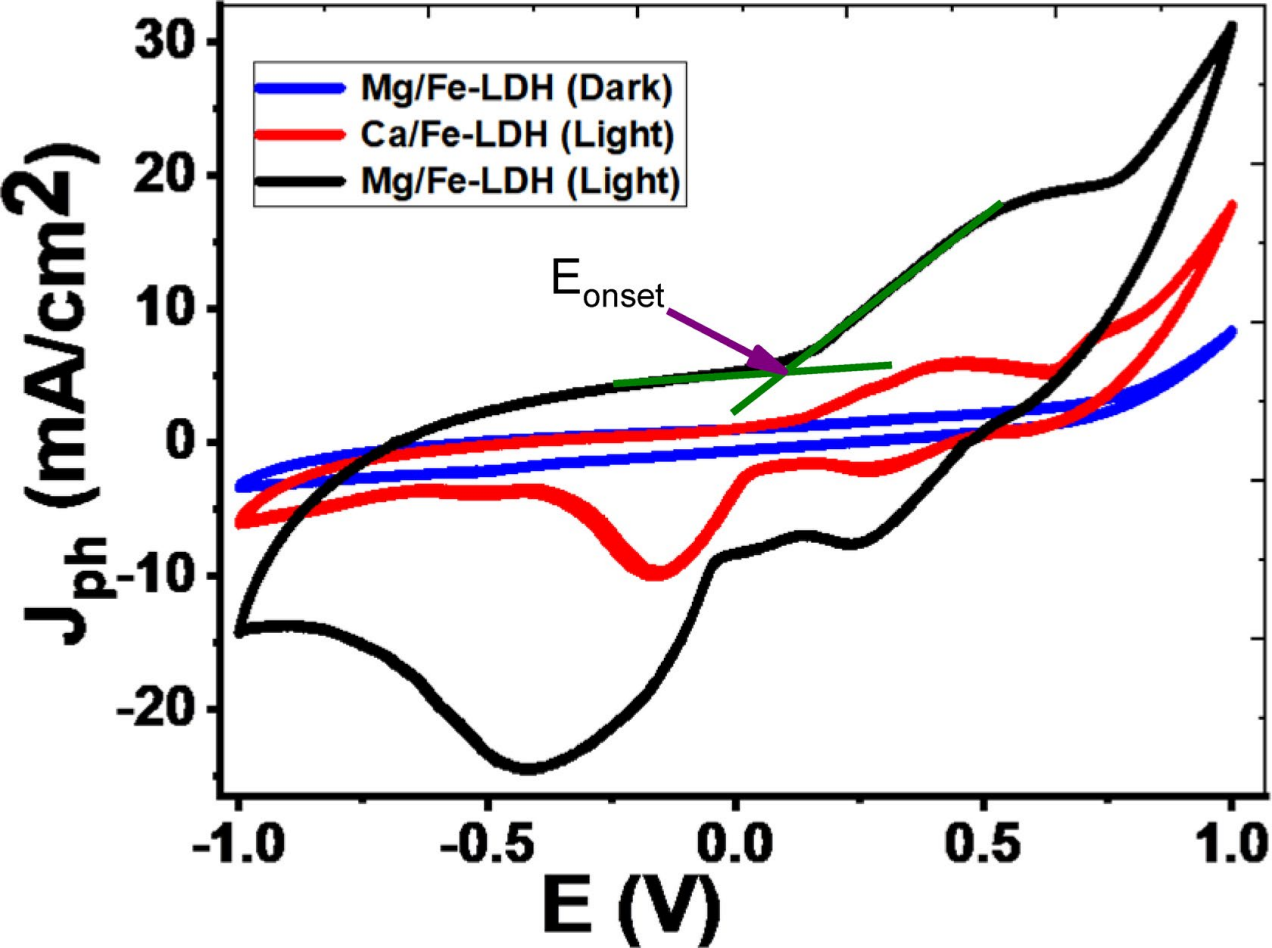
The CV of Mg/Fe-LDH in dark and light conditions and of Ca/Fe-LDH in light conditions.

Cyclic voltammetry (CV) is a well-suited electroanalytical technique to study the electrochemical performance of layered double hydroxide (LDH) photocatalysts for photoelectrochemical (PEC) hydrogen production^59^. Figure 7 shows the CV of Mg/Fe-LDH and of Ca/Fe-LDH in dark and light conditions. The superior performance of the LDH photocatalysts in the positive potential range can be correlated with a more effective Hydrogen evolution reaction (HER), which is essential for balanced water splitting and enhanced hydrogen production^60^. An important part of CV analysis is the onset voltage, which shows the potential at which a noticeable current starts^61^. This shows the start of electrochemical activity, such as the hydrogen evolution reaction (HER). The CV results showed that the Mg/Fe-LDH photocatalyst has better electrochemical behavior in light, with an onset voltage of 0.116 V. This is a lot less than what was seen for the same material when there was no light (0.71 V), which suggests that light has a strong effect on creating charge carriers that speed up the electrochemical reactions^62^. In contrast, the Ca/Fe-LDH photocatalyst had a higher onset (0.552 V) voltage even when it was subjected to light, which suggests that it would not work as well as an electrocatalyst for making PEC hydrogen. The marked difference in the onset voltages is due to photogenerated charge carriers under illumination, which reduce the energy barrier for the reaction^63^. The Mg/Fe-LDH photocatalyst’s material structure may also make it easier for hydrogen ions to attach to its surface, which leads to the release of hydrogen gas. This effect is not as strong in the Ca/Fe-LDH photocatalyst^55,64^. The CV analysis shows that the Mg/Fe-LDH photocatalyst works well as a photo-electrocatalyst. Its low onset voltage in light shows that it could be used for low-energy PEC hydrogen production.

### Efficiency of the Mg/Fe-LDH photoanode

**Fig. 8:**
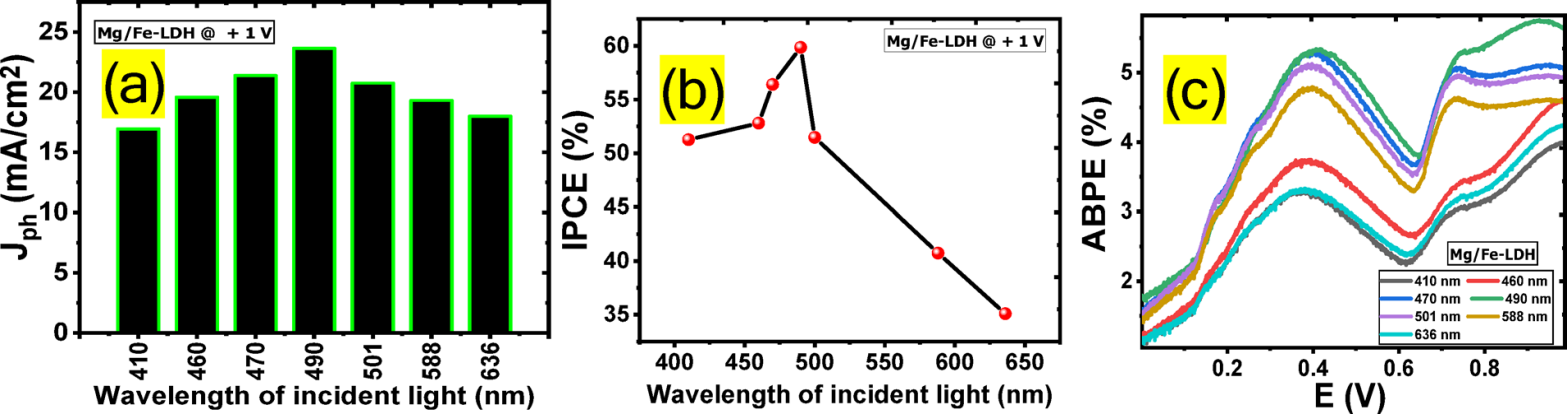
(**a**) Mg/Fe-LDH photoelectrode monochromatic light photocurrent at 1 V, (**b**) Mg/Fe-LDH photoelectrode IPCE as a function of wavelength, and (**c**) Mg/Fe-LDH photoelectrode ABPE as a function of applied voltage.

Bandpass filtering with different wavelengths between 410 and 636 nm was utilized in 0.3 M Na_2_SO_3_ at + 1 V to evaluate the Mg/Fe-LDH photoanode efficiency in the process of water oxidation for the release of hydrogen. Figure 8(a) shows that at 1 V, the photocurrent was at its lowest at 410 nm, and it was determined to be J_ph_ = 16.9 mA/cm^2^. The greatest photocurrent was measured at 490 nm, and it was determined to be J_ph_ = 23.65 mA/cm^2^. This demonstrates that the Mg/Fe-LDH photoanode is sensitive to a significant amount of sunlight and is effective at absorbing a significant amount of that sunlight in the visible spectrum. Calculating external quantum efficiency, also known as the incident photon to current conversion efficiency (IPCE), is an important way to illustrate how the Mg/Fe-LDH photoelectrode’s enhanced solar absorption and application to efficient hydrogen production from water oxidation may be applied. The IPCE for each of the different wavelengths can be estimated using Eq. (4) at an applied voltage of 1 V^65,66^.

4 $$\:\text{I}\text{P}\text{C}\text{E}\:=\:1240\times\:\frac{\text{J}\text{p}\text{h}}{({\uplambda\:}.\text{P})}\times\:\:100\:\text{\%}$$

Where λ is the wavelength of the photons that were impacted on the Mg/Fe-LDH photocatalyst and P is the illuminating light power density produced by the Xenon lamp as a function of the wavelength of the monochromatic light. The variation in IPCE as a function of wavelength is depicted in Fig. 8(b). The greatest IPCE of the Mg/Fe-LDH photoelectrode was reached when the wavelength was 490 nm, and it was about 59.85%. At 470 nm, it was 56.39%, while at 460 nm, it was 52.8%; the IPCE was 35.09% at 636 nm, which was the lowest value, which indicates that a significant percentage of incident photons are being converted into current, signifying efficient light absorption and charge generation^50^.The spectral consistency of IPCE data of Mg/Fe-LDH with the Uv-Vis absorption was mentioned in Figure S1^22^. When a low external potential is provided to the photoelectrochemical system, the electrical energy that is first supplied into the system must be removed to determine how well the photoelectrode is functioning. Alternatively, one might make use of the applied bias photon to current conversion efficiency (ABPE). The ABPE may be determined by applying Eq. (5)^67,68^.

5 $$\:\text{A}\text{P}\text{B}\text{E}={\text{J}}_{\text{p}\text{h}}\:\frac{(1.23-{\text{V}}_{\text{a}\text{p}\text{p}})}{\text{P}}\times\:100$$

V_app_ refers to the potential that is applied to the photocatalyst. The fluctuation in ABPE that occurs for the Mg/Fe-LDH as a function of the applied voltage is seen in Fig. 8(c). This occurs at a variety of wavelengths. Under conditions of monochromatic illumination, the highest possible conversion efficiency was at 490 nm and found at two different voltages: 5.75% achieved at 0.92 V, and 5.33% achieved at 0.4 V, which suggests that the energy levels in our photoanode are well-suited for efficient charge separation and transport, which is crucial for achieving high photoconversion efficiency^68^. Due to its higher IPCE and ABPE efficiencies, Mg/Fe-LDH outperforms Ca/Fe-LDH in producing hydrogen. This is because surface roughness characterization revealed that it has a larger surface area. This means that there are more active sites on the surface for electrolytes to interact, which, as expected, improves the PEC process for hydrogen production.

### Tafel parameters

**Fig. 9:**
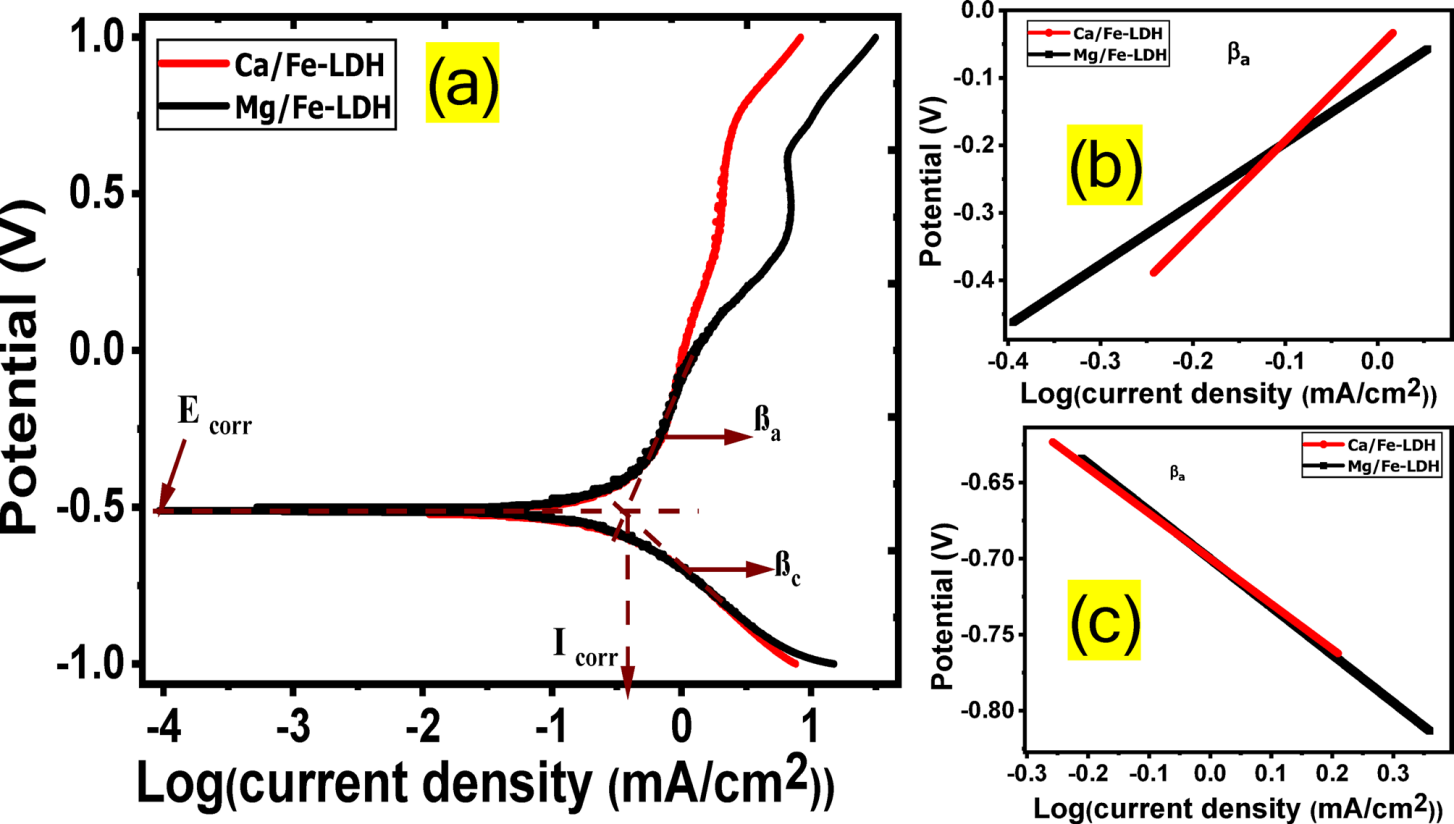
(**a**) anodic and cathodic Tafel plots of all photoelectrodes provided with the characteristic parameters, (**b**) Calculation of anodic slopes (βa), and (**c**) cathodic slopes.

The Tafel relation is used to explain HER and its rate-limiting phase. It is given by Eq. (6)^69^:

6 $${\text{V}}\,=\,\beta \,{\text{log}}\left( {{\text{Jph}}} \right)\,+\,{\text{c}}$$

Excellent HER efficiency, high current exchange rates, and low Tafel slopes are features of excellent PEC catalysts. Some photocatalysts, however, have higher current conversion rates and larger Tafel slopes, and vice versa^70^. Figure 9(a) depicts both the anodic and cathodic Tafel plots of the electrodes that are being analyzed. Essential parameters of the electrodes include the anodic and cathodic Tafel slopes (β_a_ and β_c_), corrosion potential (E_corr_), and corrosion current (I_corr_). The slopes of the linear segments of the curves in Fig. 9(b) and Fig. 9(c) determine the β_a_ and β_c_ values for the electrodes under study^71^. The values of E_corr_, I_corr_, β_a_, and β_c_ for all electrodes are presented in Table 1. The βa value decreases from 1.37 V/dec for Ca/Fe-LDH to 0.9 V/dec for Mg/Fe-LDH. The βc value rose from ‒0.297 V/dec for Ca/Fe-LDH to ‒0.31 for Mg/Fe-LDH. Tafel PEC slopes provide PEC reaction processes and rate-limiting steps. With a Tafel slope of roughly 30 mV/decade, the Volmer-Tafel mechanism illustrated in Eqs. (7)-(9) predominates during rate-limiting recombination. When the Tafel slope is roughly 40 mV/decade and PEC desorption is rate-limiting, the Volmer-Heyrovsky hydrogen evolution mechanism is most likely to be in charge^72^. A slope of ~ 120 mV/decade on the Tafel reveals that reaction pathways rely on the surface laden with adsorbed hydrogen. A β_c_ number represents the overpotential required to enhance the HGR rate by a factor of 10. The low Tafel slopes of the Mg/Fe-LDH photoelectrode and the low energy of the band gaps resulted in low overpotentials for the photoelectrodes. This is because only a small quantity of energy is required to improve HGR performance, and vice versa. While the corrosion rate may be found at I_corr_, the corrosion tendency of the solution can be found at E_corr_. Table 1 shows that the Mg/Fe-LDH photoelectrode exhibited nobler behavior than the Ca/Fe-LDH, with an E_corr_ of ‒0.51 V, which is lower. One can evaluate the photoelectrode’s resistance to corrosion based on its I_corr_. Table 1 illustrates that Mg/Fe-LDH’s I_corr_ is 0.392 mA/cm^2^ less than Ca/Fe-LDH’s 0.4677 mA/cm^2^. We were able to compute the polarization resistance (Rp in Ω.cm^2^) using the Stern-Geary equation and the straight part of the curves at Ecorr by utilizing the following relation: R_p_ = β_c_ β_a_ / [2.303 I_corr_ (β_c_ + β_a_)]. Table 2 contains a list of the measured R_p_ for every electrode. For Ca/Fe-LDH, R_p_ is 2.2 Ω.cm^2^, whereas for Mg/Fe-LDH, it is 0.5 Ω.cm^‒2^.

7 $$\:{\varvec{H}}_{\left(\varvec{a}\varvec{q}\right)}^{+}+\varvec{S}+{\varvec{e}}^{-}\to\:\varvec{S}-\varvec{H}\:\left(\varvec{a}\varvec{d}\varvec{s}\varvec{o}\varvec{r}\varvec{p}\varvec{t}\varvec{i}\varvec{o}\varvec{n}\:\varvec{a}\varvec{n}\varvec{d}\:\varvec{d}\varvec{i}\varvec{s}\varvec{c}\varvec{h}\varvec{a}\varvec{r}\varvec{g}\varvec{e}\:\varvec{o}\varvec{f}\:\varvec{p}\varvec{r}\varvec{o}\varvec{t}\varvec{o}\varvec{n}\right)$$

8 $$\:\varvec{S}-\varvec{H}+{\varvec{H}}_{\left(\varvec{a}\varvec{q}\right)}^{+}\to\:\varvec{S}-\varvec{H}-{\varvec{H}}^{+}\:\left(\varvec{a}\varvec{d}\varvec{s}\varvec{o}\varvec{r}\varvec{p}\varvec{t}\varvec{i}\varvec{o}\varvec{n}\:\varvec{a}\varvec{n}\varvec{d}\:\varvec{a}\varvec{n}\varvec{o}\varvec{t}\varvec{h}\varvec{e}\varvec{r}\:\varvec{p}\varvec{r}\varvec{o}\varvec{t}\varvec{o}\varvec{n}\:\varvec{o}\varvec{n}\:\varvec{t}\varvec{h}\varvec{e}\:\varvec{s}\varvec{a}\varvec{m}\varvec{e}\:\varvec{s}\varvec{i}\varvec{t}\varvec{e}\right)$$

9 $$\:\varvec{S}-\varvec{H}-{\varvec{H}}^{+}+{\varvec{e}}^{-}\:\to\:\:{\varvec{H}}_{2}\left(\varvec{g}\right)+\varvec{S}\:\left(\varvec{d}\varvec{i}\varvec{s}\varvec{c}\varvec{h}\varvec{a}\varvec{r}\varvec{g}\varvec{e}\:\varvec{a}\varvec{n}\varvec{d}\:\varvec{d}\varvec{e}\varvec{s}\varvec{o}\varvec{r}\varvec{p}\varvec{t}\varvec{i}\varvec{o}\varvec{n}\:\varvec{o}\varvec{f}\:{\varvec{H}}_{2}\right)$$

The Volmer–Heyrovsky mechanism from above equations is expected to manifest at catalytic interfaces where the availability of electroactive surface sites is significantly diminished, resulting in a separation between adjacent sites that exceeds the van der Waals radius of two adsorbed hydrogen atoms, thereby precluding the occurrence of the Tafel mechanism. In this scenario, the sole route for the evolution of H_2_ is the Volmer–Heyrovsky pathway, illustrated below^73^. The limited number of electroactive sites on the electrocatalyst’s surface necessitates that the hydrogen evolution reaction (HER) proceeds via the mechanism to produce H_2_^73^.

**Table 2:** Values of corrosion and Tafel parameters of all photoelectrodes.

photocatalyst	E_Corr_ (V)	I_Corr_ (mA.cm^‒2^)	β_a_ (V/dec)	*R* ^2^	β_c_ (V/dec)	*R* ^2^	Rp (Ωcm^2^)
Ca/Fe-LDH	‒0.52	0.4677	1.37	0.96	‒0.297	0.99	2.21
Mg/Fe-LDH	‒0.51	0.392	0.9	0.96	‒0.31	0.99	0.5

Table 3 presents a comparison of performance indicators, illustrating the superior performance of prepared electrodes with previously published literature^74,75^. The results indicate that our Mg/Fe-LDH photoelectrode displays better efficiency and photogenerated current density compared to the electrodes described earlier. These findings underscore the enormous potential of this photoelectrode in the realms of photocatalysis and sustainable energy production.

**Table 3:** Comparison of LDHs hydrogen generation parameters of previous investigations utilizing nanostructured LDHs and the current work.

Electrocatalyst	Electrolyte	Jph (mA/cm^2^)	IPCE (%)	ABPE (%)	H_2_ moles	Ref.
T-LDH/PbI2 NC	0.3 M KOH	53.27 @‒1 V	83 @307 nm	-	107.53 mmole/h.cm^2^	^74^
BiVO4/Ni0_0.5_Fe_0.5_-LDH	0.5 M Na_2_SO_4_	1.21 @1.23 V vs. Ag/Agcl	37.5	-	-	^75^
Ni-Fe-LDH/ZnO	1 M KOH	1.7 @ 2 V vs. Ag/AgCl	82 @380 nm	-	19 mmol/ h.cm^2^	^76^
Co-LDH	0.5 M Na_2_SO_4_	4.67 @ 0.8 V vs. SCE	1.31 @365 nm	-	-	^77^
CdS/ZnCr-LDH	0.1 M Na_2_SO_3_	-	42.6 @UV-Vis.	-	2164 µmol/h.g	^78^
Co-Mo LDH	1 M KOH	10 @1.35 V	-	-	-	^79^
Graphene/CoAl LDH @BiVO_4_	0.1 M Na_2_SO_3_	2.13 @1.23 V	52 @400 nm	-	-	^80^
BiVO_4_/CdS/NiCo-LDH	0.5 M Na_2_SO_3_	2.72 @1.23 V	30.25 @420 nm	1.24 @ 0.62 V	-	^81^
Mg/Fe-LDH	0.3 M Na_2_SO_3_	31.55 @1 V	59.85 @490 nm	5.33 @0.4 V	2542.36 mmole/h.cm^2^	This work

**Fig. 10:**
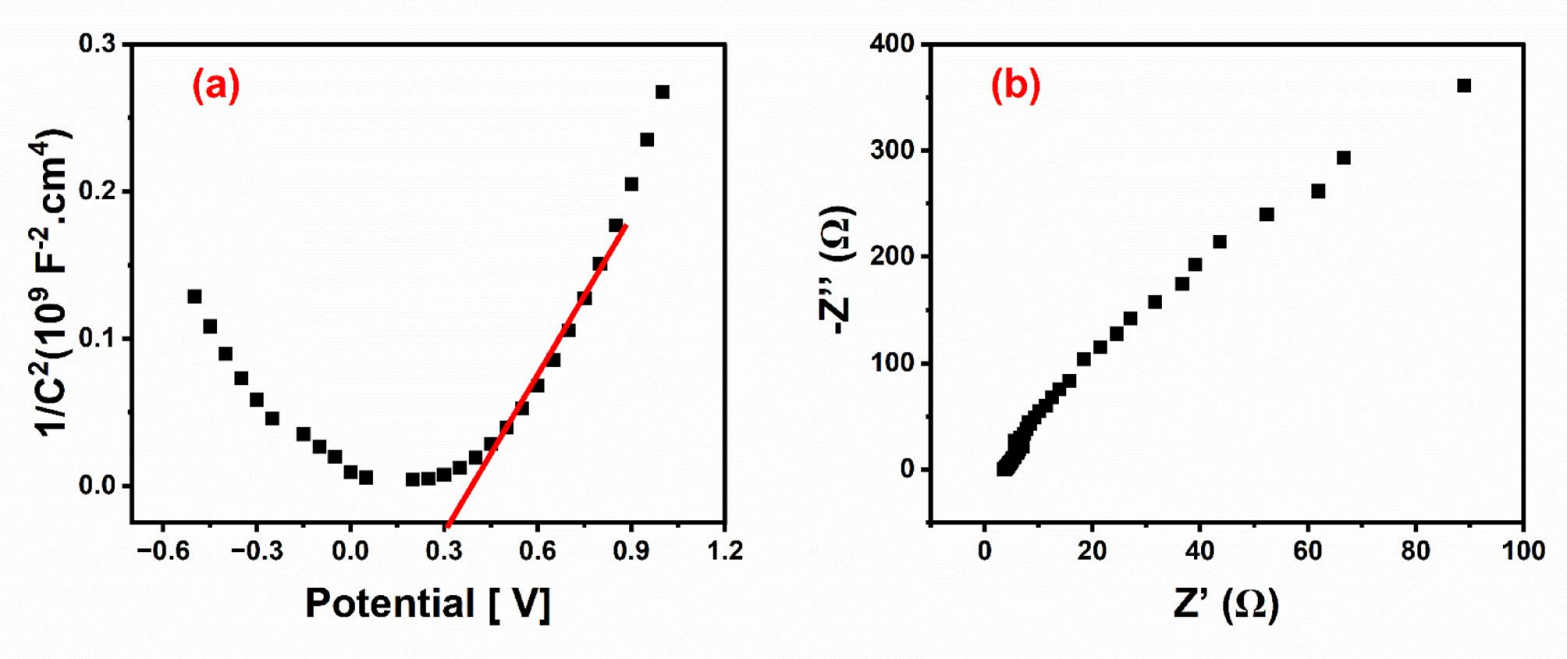
**a**) Mott-Schottky plot, and **b**) Nyquist impedance plots for Mg/Fe-LDH.

Mott-Schottky measurements were conducted by varying the applied potential from − 0.5 V to 1 V at a specified frequency of 10 kHz to ascertain the kind of semiconductor; a positive slope indicates n-type, while a negative slope indicates p-type. The flat-band potential (E_FB_) and the type and concentration of charge carriers in the Mg/Fe-LDH photoanode can be ascertained using the Motte-Schottky Eqs. (10)-(11)^82^.

10 $$\:{C}_{sc}^{-2}=\pm\:\frac{2}{e\epsilon\:{\epsilon\:}_{0}{N}_{d}}({E}_{appl}-{E}_{FB}-\frac{kT}{q})$$

11 $$\:{N}_{d}=\left(\frac{2}{e\epsilon\:{\epsilon\:}_{0}}\right){\left[\frac{d({C}^{-2}}{d{E}_{appl}}\right]}^{-1}$$

C_sc_ denotes the capacitance of the space-charge region, e represents the electron charge, ε_0_ signifies the dielectric vacuum permittivity, ε_r_ indicates the dielectric constant of the semiconductor, N_d_ refers to the donor density, A is the surface area of the electrode in contact with the electrolyte, E_Appl_ is the applied potential on the reference electrode, k is Boltzmann’s constant, T is the temperature, and c is a constant that varies with the type of reference electrode employed^83^.

Figure 10 (a) show that The slope of the linear component of the Mott-Schottky plots of Mg/Fe-LDH was utilized to acquire the (d(C^− 2^)/dV) values. According to calculations, the slope of Mg/Fe-LDH is 0.37 × 10^9^. Since Mg/Fe-LDH has a positive Mott–Schottky slope, it is an n-type semiconductor with electrons as the major carrier. The Mott–Schottky plots are used to determine the carrier densities. The results show that the donor density of Mg/Fe-LDH is 9.502 × 10^18^cm^3^. The higher photocurrent for Mg/Fe-LDH can be attributed to the increased donor density with Hydrogen vacancies on the surface. The larger donor density might boost the conductivity, and then the surface electrons can be promptly transferred, which may reduce the recombination of photoexcited holes with electrons^84^, and prefer the − 0.5 to 1 V. The flat band potentials of the electrode (the intersection of the tangent and X-axis) are determined to be about 0. 31 V and the conduction and valence bands are 0.42 eV and 4.86 eV, respectively. (EIS) The Nyquist diagram is a useful method for characterizing the interfacial characteristics in PEC systems. In Fig. 10 (b), the Nyquist diagram is linear with Capacitance and includes the charge separation and transfer behaviour between the semiconductor and PEC electrolyte interfaces^85^. Mg/Fe-LDH has an ionic conductivity is 1.43 mS cm-1.

### Theoretical analysis

**Fig. 11:**
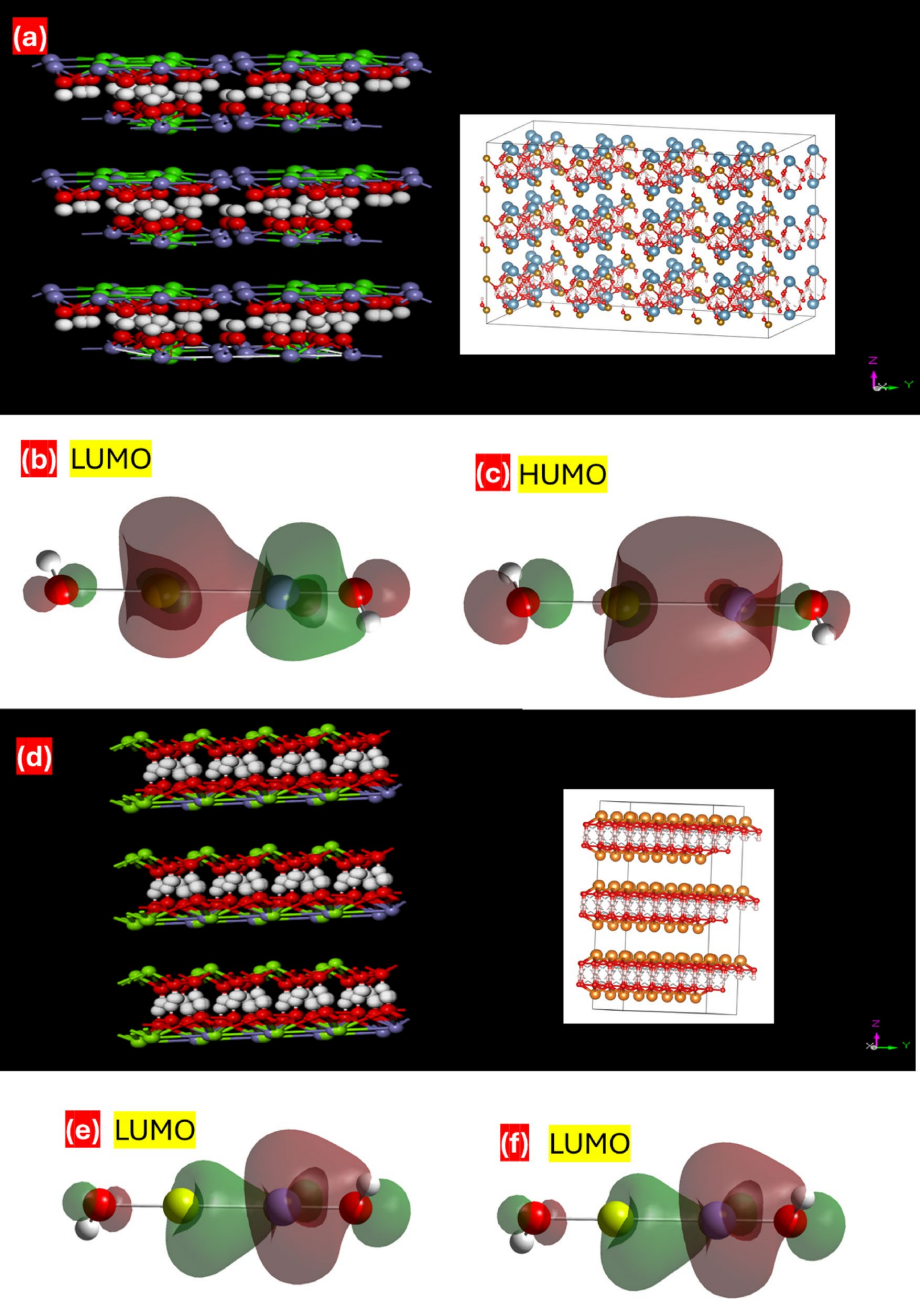
(**a**) Structure of Ca/Fe-LDH, (**c**), (**d**) LUMO and HUMO of Ca/Fe-LDH, and, (**d**) Structure of Mg/Fe-LDH and (**e**),(**f**) LUMO and HUMO of Mg/Fe-LDH.

Study Ca/Fe and Mg/Fe-LDH simulations for use in PEC water splitting applications. The HOMO and LUMO gap energy for Ca/Fe and Mg/Fe-LDH are illustrated by the frontier molecular orbitals (FMOs) for HOMO and LUMO.These are key factors in calculations involving quantum chemistry that portray systems under research at the microscopic level. Strong indicators of an interaction system’s electronic characteristics include the HOMO-LUMO energy gaps (Eg), the lowest unoccupied molecular orbital (LUMO), and the highest occupied molecular orbital (HOMO). The border orbitals, generally referred to as the HOMO and LUMO, are key variables that reveal qualitative data on the excitation properties of simulated materials. In the meantime, a convenient instrument for finding out the chemical reactivity of molecules interacting. Furthermore, a molecule’s chemical stability is determined by its hardness; a molecule with a lower chemical stability is softer and more reactive^86^.

The gap energy values for Ca/Fe and Mg/Fe-LDH are 2.755 eV, and 2.566 eV, respectively illustrated in (Fig. 11) (a) to (f). The chemical reactivity of Mg/Fe-LDH is higher than that of for Ca/Fe-LDH because the former has a smaller gap energy and the largest electronic transitions. These results further support the data gained from the experimental technique, revealing the effect of the chemical composition of Mg/Fe LDH and Ca/Fe LDH on energy stability for PEC water splitting. Structural modifications, especially the replacement of Mg for Ca, affect the ESP properties and Energy gap, which lowers the energy, which can boost charge separation and transport efficiency throughout the water splitting process. gap values. Subsequently, the electronic structures of the optimized geometry were estimated to explicate whether the two LDH are suitable for PEC water splitting. A greater dipole moment is discovered for Mg/Fe-LDH (2.250836 Debye) than Ca/Fe -LDH(0.422814 Debye).

The quantum molecular descriptors applied in the DFT computation are chemical-reactivity descriptors (µ, ˕, σ, ω, ∆N) that describe the utility of Ca/Fe and Mg/Fe-LDH in PEC water splitting. The qualities that are most frequently employed include the maximum charge transfer index (∆N), softness (σ), global hardness (η), chemical potential (µ), and electrophilicity index (ω)^87^. Global hardness, commonly known as a molecule’s hardness (η), is directly related to how resistant a molecule is to changes in electrical distribution and has been demonstrated to be effective in explaining chemical reactions. On the other side, softness is the inverse of global hardness (σ = 1/η). Electronegativity (χ) is a measure of an element’s capacity to absorb electrons and produce negative ions during the chemical process of a delivery system^88^. The electrophilicity index (ω) analyzes a molecule’s reactivity by assessing its capacity to absorb electrons, whereas the chemical potential (µ) shows the direction of electron flow from the higher µ to the lower µ until the chemical potential is equilibrated^89^. The mathematical relationship between the quantum molecular descriptors and electrical properties is illustrated by Eqs. (12)–(17).

12 $$\:\eta\:\:=\frac{I-A}{2}$$

Where I(ionization potential ) = -E HUMO and A(electron affinity) = - LUMO

13 $$\:{\upmu\:}\:=\frac{EHOMO\:+\:ELUMO}{2}$$

14 $$\:\chi\:\:=\:\frac{I\:+\:A}{2}$$

15 $$\:\omega\:\:=\:\frac{{{\upmu\:}}^{2}}{2\eta\:}$$

16 $$\:\varDelta\:Nmax\:=\:-\frac{{\upmu\:}}{\eta\:}$$

17 $$\:\varvec{\sigma\:}\:=\:\frac{1}{\eta\:}$$

The targeted region’s softness, hardness, electronegativity, and electrophilic index can all be related to the molecular toxicity, polarizability, structural stability, and reaction rate of any chemical molecule in the biological system. These values are 0.726, 1.377, 7.498, and 4.1315 for Ca/Fe and Mg/Fe-LDH, respectively, and 0.7794, 1.283, 9.805, and 4.988 at Mg/Fe-LDH. Additionally, the maximal charge transfer indices (∆N) for Ca/Fe-LDH and Mg/Fe-LDH were 3 and 3.909, respectively.

The reactivity properties reported in and the overall stability of these compounds are supported by the substantially bigger gap between HOMO and LUMO. The enhanced hardness and decreased chemical softness support this. The chemical potential (µ) is the propensity of an electron to escape from a stable molecule. The complex is stable and unable to break down spontaneously into its constituent atoms due to its negative chemical potential. Examine the energy difference between Ca/Fe and Mg/Fe-LDH to evaluate a molecule’s capability. It was revealed that the energy gap ranges between 2.755 eV and 2.566 eV, reflecting changes in the electron distribution of the molecule. According to this, Mg/Fe-LDH worked better for PEC water splitting to generate hydrogen.

**Fig. 12:**
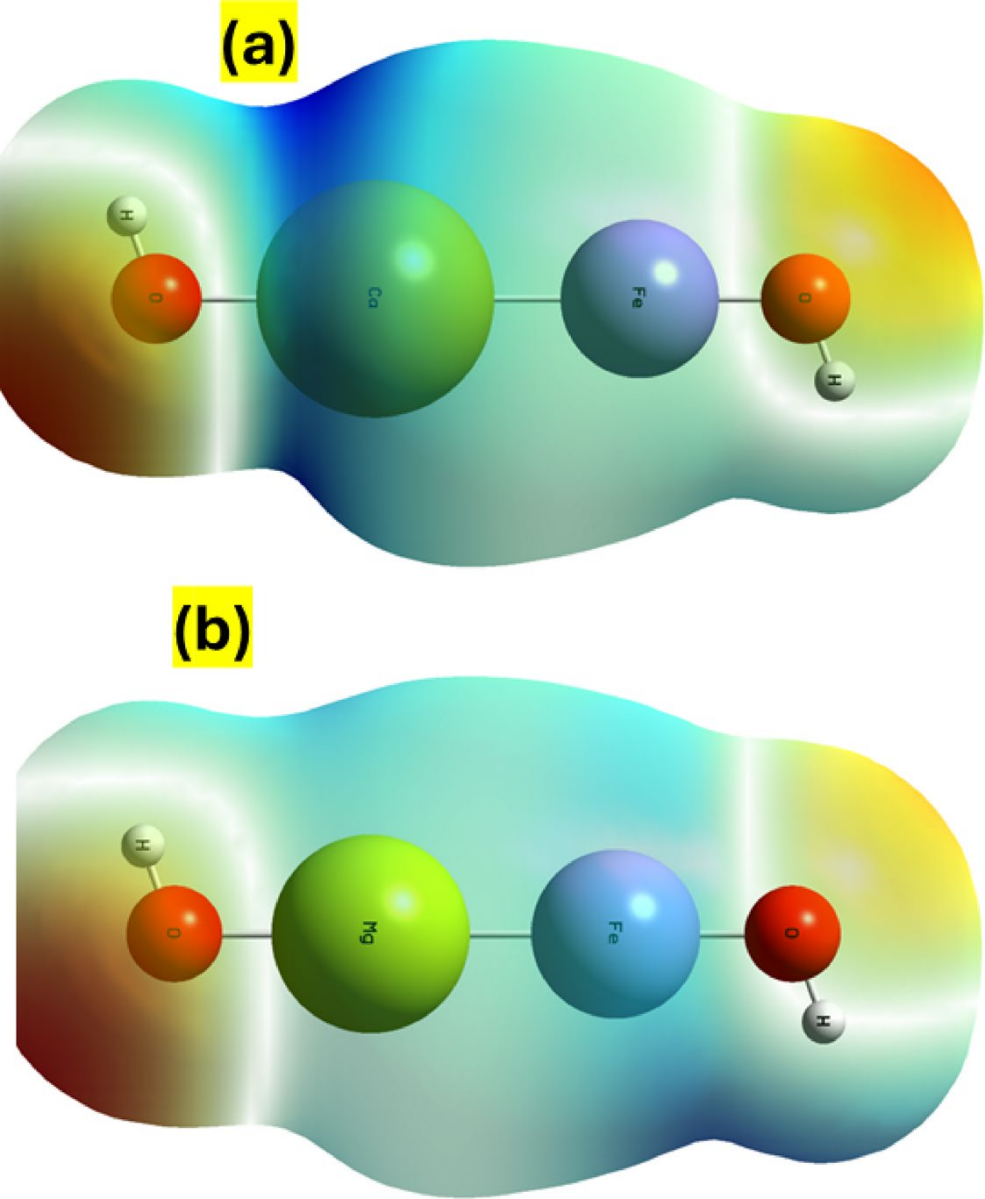
(**a**) Molecular electrostatic potentials (MEP) plot of Ca/Fe-LDH, and (**b**) Molecular electrostatic potentials (MEP) plot of Mg/Fe-LDH.

When the electrophilic and nucleophilic activities occur in the molecule, the electrostatic potential (ESP) graphing in the iso-surface plot and the electron density mapping in the MEP plot have been analyzed for the charge distribution concepts. Different colors are utilized to illustrate the Ca/Fe-LDH and Mg/Fe-LDH total density mapping. Chemically active sites and atomic-level comparative reactivity have been utilized to depict these molecular graphs.

Figure 12(a) displays the surface plots of molecule electrostatic potentials for Ca/Fe and Mg/Fe-LDH, a significant computation utilized in the analysis of intermolecular characterization and photocatalytic processes. The spatial distribution of MEPs is graphically shown as the chemical activities linked with a chemical reaction and robust binding to the active sites. MEP is integrally linked to the chemical behavior, electronegativity, and dipole moment of Ca/Fe and Mg/Fe-LDH. These graphs depict the molecule’s features in terms of electron densities (ED) and illustrate its relative polarity^90^. MEPs surface maps have been traced using different colors based on the electrostatic significance: red shows the molecule’s highest -ve potential, blue its most + ve potential, and green its zero potential regions^91^. The order of MEP increases on surface mapping/arrays is red, orange, yellow, green, and blue. In this situation, the blue color signifies electrophilic reactivity while the red hue denotes nucleophilic reactivity. Although the positive MEP demonstrates the repulsion of the proton/cation in the blue zone owing to the atomic nuclei areas as the low concentration of ED in the molecule, the negative MEP displays the attraction of a proton/light cation in the red region due to concentrated ED in the molecule^92^. In Fig. 12(a), the Ca-OH region is represented by the blue region, and the positive potential demonstrates that the proton/cation area—where electrophilic reaction activity is found—is repelled. However, a largely green and yellow zone with zero and medium electric potential regions surrounds the other atoms. These graphs reflect the molecule’s characteristics in terms of electron densities (ED) and indicate its relative polarity. MEPs surface maps have been traced using different colors based on the electrostatic significance: red shows the molecule’s highest -ve potential, blue its most + ve potential, and green its zero potential regions. MEP rises in the following order on surface mapping/arrays: red, orange, yellow, green, and blue. Here, the electrophilic reactivity is indicated by the blue color, whereas the nucleophilic reactivity is exhibited by the red hue. While the positive MEP demonstrates the repulsion of the proton/cation in the blue zone due to the atomic nuclei areas as the low concentration of ED in the molecule, the negative MEP displays the attraction of a proton/light cation in the red region due to concentrated ED in the molecule^93^. The positive potential depicts the repulsion of the proton/cation area, which is where electrophilic reaction activity is located, and the Ca-OH region is positioned with the blue region in Figure. 12(a). The other atoms, meanwhile, are surrounded by a zone that is primarily green and yellow and has zero and medium electric potential zones. However, the area of Mg-OH between red and yellow in figure. 12(b) The proton/cation area, which is the site of nucleophilic reaction activity, is drawn to this -ve potential, as represented by the red hue of the oxygen between Ca, Mg, and Fe. In order to identify probable active spots for the photocatalytic activity, the charge density, electrostatic potential (ESP) surface, and Bader charge were then computed. Their increased electronegativity is intimately linked to the charge densities, as demonstrated in Figure (12). Sites in Ca/Fe-LDH had a lower electrostatic potential value of − 0.77 eV than in Mg/Fe-LDH of − 0.93 eV, according to the ESP analysis shown in Figure. 12(b). This reveals that Ca/Fe-LDH has a stronger nucleophilicity than Mg/Fe-LDH. As a result, the Mg/Fe-LDH often has the best current density for PEC water splitting’s hydrogen generation, which necessitates a narrow energy gap and high ESP.

## Conclusion

This study successfully demonstrates a simple method for preparing Mg/Fe and Ca/Fe layered double hydroxides as novel composites for hydrogen photocatalytic production. The Mg/Fe-LDH material shines in performance, boasting a nearly fourfold higher photocurrent density (31.55 mA/cm²) at 1 V compared to its Ca/Fe counterpart (8.2 mA/cm²). Remarkably, Mg/Fe-LDH also exhibits an impressive IPCE of 59.85% at 470 nm and an ABPE of 5.75% at 0.92 V. Furthermore, Mg/Fe-LDH offers notable stability and high resistance to corrosion. These findings provide valuable insights for designing efficient photocatalytic materials based on Mg/Fe-LDH for sustainable hydrogen generation using solar energy. Furthermore, the experimental results were Confirmed by DFT calculations, which enhanced the understanding of the band gap structure and ESP characteristics of LDH nanomaterials.

## Supplementary Information

Below is the link to the electronic supplementary material.

Supplementary Material 1

## Data Availability

The data will be available upon request, F.M. and O.M.
