# Supplementary material for: Experimental and theoretical insights into LDH based on iron for photoelectrochemical water splitting
Source: Sci Rep. 2025 Oct 14;15:35801. doi: 10.1038/s41598-025-17648-0 (PMC12521562; doi:10.1038/s41598-025-17648-0)
Supplement: Supplementary file 1 — Supplementary Material 1 [file 41598_2025_17648_MOESM1_ESM.docx]

**supplementary file**

**Experimental and Theoretical Insights into LDH based on Iron for Photoelectrochemical Water Splitting**

**Fatma Mohamed**^1,^**^2 *^, Omnia M. Salem**^1,^**^2*^**, **Khaled Abdelkarem**^1^, **Mohamed Shaban**^1,3^, **Ashour M. Ahmed**^1,4^

^1^Nanophotonics and Applications Lab, Physics Department, Faculty of Science, Beni-Suef University, Beni-Suef 62514, Egypt; [f_chem2010@yahoo.com; omniamohamed_pg@science.bsu.edu.eg](mailto:f_chem2010@yahoo.com;%20omniamohamed_pg@science.bsu.edu.eg) ; [oldfighter.khaled123@gmail.com](mailto:oldfighter.khaled123@gmail.com) ; [mssfadel@aucegypt.edu](mailto:mssfadel@aucegypt.edu) ; [asmmohamed@imamu.edu.sa](mailto:asmmohamed@imamu.edu.sa) .

^2^Materials Science Research Laboratory, Chemistry Department, Faculty of Science, Beni-Suef University, Beni-Suef, Egypt; [f_chem2010@yahoo.com](mailto:f_chem2010@yahoo.com) [.omniamohamed_pg@science.bsu.edu.eg](mailto:.omniamohamed_pg@science.bsu.edu.eg)

^3^Department of Physics, Faculty of Science, Islamic University of Madinah, P. O. Box: 170, AlMadinah Almonawara 42351, Saudi Arabia; [mssfadel@aucegypt.edu](mailto:mssfadel@aucegypt.edu).

^4^Physics Department, College of Science, Imam Mohammad Ibn Saud Islamic University (IMSIU), Riyadh, 11623, Saudi Arabia. [asmmohamed@imamu.edu.sa](mailto:asmmohamed@imamu.edu.sa)

**Table S1: The SCF Parameters of the Mg/Fe-LDH and Ca/Fe-LDH**

|  | **E(RB3LYP) Hartree** | **A.U.** | **NFock** | **Conv** | **-V/T** |
| --- | --- | --- | --- | --- | --- |
| **Mg/Fe-LDH** | **-1615.38258873** | **after 27 cycles** | **27** | **0.91D-08** | **2.0011** |
| **Ca/Fe-LDH** | **-2092.85826000** | **after 27 cycles** | **27** | **0.56D-09** | **2.0010** |

**Table S2: unit cell parameter of the Mg/Fe-LDH and Ca/Fe-LDH**

|  | **A “Angstrom”** | **B “Angstrom”** | **C “Angstrom”** | **α “degree”** | **β “degree”** | **γ “degree”** |
| --- | --- | --- | --- | --- | --- | --- |
| **Mg/Fe-LDH** | **6.968** | **12.973** | **23.813** | **90** | **90** | **120** |
| **Ca/Fe-LDH** | **20** | **40** | **26.246** | **90** | **90** | **90** |

**

**

**Figure S1**. Comparative IPCE plots of Mg/Fe-LDH electrode within 300-700 nm, represented by lines with the corresponding absorption spectra of Mg/Fe-LDH .
